# Environmental Regulation, Two-Way Foreign Direct Investment, and Green Innovation Efficiency in China’s Manufacturing Industry

**DOI:** 10.3390/ijerph15102292

**Published:** 2018-10-19

**Authors:** Zhijun Feng, Bo Zeng, Qian Ming

**Affiliations:** School of Economic and Management, Dongguan University of Technology, Dongguan 523808, China; fengzj@dgut.edu.cn (Z.F.); 2017102@dgut.edu.cn (Q.M.)

**Keywords:** environmental regulation, two-way FDI, green innovation efficiency, industry heterogeneity

## Abstract

This paper adopts 2009 to 2015 panel data from 27 manufacturing industries in China. A Super-SBM model is used to measure the green innovation efficiency (GIE) of China’s manufacturing industry. A panel data model is then built to systematically examine the impact of environmental regulation (ER) and two-way foreign direct investment (FDI) on the GIE of China’s manufacturing industry under a unified analysis framework. The results are as follows: (1) the overall level of the green innovation efficiency in China’s manufacturing is low, and there is still great potential for improvement. Considering industry heterogeneity, the green innovation efficiency of patent-intensive manufacturing is significantly higher than that of non-patent-intensive manufacturing; (2) in terms of the whole manufacturing industry, ER and the interaction between ER and outward foreign direct investment (OFDI) have significantly negative effects on GIE, OFDI has significantly positive effects on GIE. (3) when considering industry heterogeneity, for patent-intensive manufacturing, ER and the interaction between ER and inward foreign direct investment (IFDI) have significantly negative effects on GIE, while IFDI has significantly positive effect on GIE. For non-patent-intensive manufacturing, ER and the interaction between ER and OFDI have significantly negative effects on GIE, while IFDI and the interaction between ER and IFDI have significantly positive effects on GIE.

## 1. Introduction

The manufacturing industry is the core of China’s real economy and is thus of great significance to the industrialization and economic development in the country [1,2]. However, the extensive development model adopted by China’s manufacturing industry for a long time, has not only caused it to be at the bottom of the international industrial chain, but also caused serious ecological problems [3,4]. In 2016, manufacturing industry accounted for 29.9% of China’s GDP, consumed about 57% of the China’s energy consumption, and accounted for over 50% of China’s total CO_2_ emissions [5]. The gray wall covering millions of square kilometers across the country from Beijing to Hong Kong serves as example, according to the satellite haze map from NASA [2]. In the past few years, China’s growing energy demand and increasingly serious environmental pollution emissions have caused widespread international concern and concern [6,7]. Therefore, the “Made in China 2025” plan clearly stated that it will speed up the green transformation and upgrading of the manufacturing industry. Innovation is the core driving force for industrial transformation and upgrading, and green innovation has become the key to achieving a green transformation in the manufacturing industry. 

With the constant advancement of the “One Belt and One Road” initiative and the free trade zone strategy, China has gradually played an increasingly important role in the international capital arena in the dual role of host country and investment country. According to data from China’s Ministry of Commerce, the actual utilization of inward foreign direct investment (IFDI) in China increased from 53.5 billion dollars to 126 billion dollars from 2003 to 2016, with an average annual growth rate of 7.13%. Accordingly, China’s non-financial outward foreign direct investment (OFDI) jumped from 2.8 billion dollars to 170.1 billion dollars, with an average annual growth rate of 42.01%. According to the literature, promoting green innovation in manufacturing industry, through international direct investment may be effective [8,9]. Foreign direct investment coming into China, IFDI not only provides strong financial support for China’s manufacturing industry to carry out green innovation activities, but it is also one of the main sources of access to advanced green innovation resources. OFDI is an important channel for international R&D spillovers, enterprises actively acquire advanced technology in the host country with intensive technology resources through OFDI, and then digest and absorb them for their own use, and then transfer them to the parent company in the home country through reverse technology spillover [10,11,12]. In fact, the motivation to promote China’s technological progress or green innovation performance through OFDI’s reverse technology spillover has long been confirmed by many studies [13,14,15,16,17].

However, green innovation has the characteristic of “double externality” [18,19], as enterprises lack clear economic incentives to actively implement green innovation. In this case, it is necessary for the government to formulate relevant environmental regulations [20,21]. However, under current economic globalization, environmental regulation is a double-edged sword. China’s increasingly strict environmental regulation standards will inevitably have a complex impact on international direct investment. Therefore, under such circumstances, environmental regulation is inevitably an important factor affecting the relationship between two-way FDI (IFDI and OFDI) and green innovation in China’s manufacturing industry. Ignoring the environmental regulatory factor, and considering only the relationship between two-way FDI and green innovation in China’s manufacturing industry, may lead to some misleading conclusions. Therefore, what is the impact mechanism of environmental regulation and two-way FDI on green innovation in China’s manufacturing industry? Is there any industry heterogeneity in the impact of environmental regulation and two-way FDI on green innovation in China’s manufacturing industry? In the process of two-way FDI affecting green innovation in China’s manufacturing industry, what role does environmental regulation play? The answers to the above questions are the focus of this paper. Based on panel data of 27 manufacturing industries in China from 2009 to 2015, this paper uses the Super-SBM model to measure the green innovation efficiency of China’s manufacturing industry, and then empirically analyses the impact of environmental regulation and two-way FDI on the green innovation efficiency in China’s manufacturing industry.

The rest of the paper is organized as follows: Section 2 presents a literature review, theoretical analysis, and hypothesis. Section 3 describes model setting, variable selection and description, sample selection and data source. Section 4 shows empirical results and analysis. Finally, Section 5 presents research conclusions, policy recommendations, and future research prospects. 

## 2. Literature Review and Research Hypothesis

### 2.1. Literature Review

#### 2.1.1. The Relationship between Environmental Regulation and Green Innovation

The existing research actively explores the relationship between environmental regulation and green innovation. There are four main viewpoints: environmental regulation promotes green innovation, environmental regulation inhibits green innovation, environmental regulation and green innovation show a “U-shaped” relationship, and the role of environmental regulation on green innovation is uncertain. 

The first viewpoint is that environmental regulation can transform the direction of technological progress and development, which helps industry to embark on the track of green innovation. Porter and Van [22] found that environmental regulation promotes green innovation in enterprises. Ambec and Barla [23] believed that if the government’s environmental regulation intensity and economic development level can be balanced, the implementation of environmental regulation policies can effectively stimulate technological innovation. Arimura and Sugino [24] used data for 20 years of environmental policy, environmental research and development expenditures, environmental performance and business performance in seven OECD countries; they found that there is a significantly positive relationship between environmental regulation and green innovation investment. Horbach [25] demonstrated the driving effect of environmental regulation on corporate green innovation using panel data from German manufacturing enterprises. Kammerer [26]; through empirical research on 92 electronics enterprises in Germany, found that environmental regulation has a positive correlation with green innovation. Kneller and Manderson [27] used the dynamic panel data model developed by the generalized moment method to analyze the impact of environmental regulation on green innovation; they found that the pressure brought by pollution control will promote enterprises to carry out green innovation. Kesidou and Demirel [28] concluded that environmental regulation promotes green technology innovation. Acemoglu et al. [29] used the two-sector-oriented technology advancement model to study the impact of different environmental regulation tools on green technology advancement and pollution-based technological progress. They found that when the clean production sector and the non-clean production sector can be fully replaced, through temporary environmental regulation tools can shift innovation investment to the cleaner production sector and achieve green technology advancement. Calel and Dechezlepretre [30] investigated the impact of the EU Emissions Trading System (EUETS) on technology changes. They found that the EUETS has increased low carbon innovation in regulated enterprises by up to 10%. Jia and Zhang [31] found that environmental regulation significantly inhibits the impact of non-green technical knowledge stocks within and outside the region, which promotes both green and non-green innovation. Castellacci and Lie [32] investigated different types of eco-innovations in South Korea, and the results show that environmental taxes and regulations are identified to be more important drivers of technological change for pollution-reducing firms.

The second viewpoint is that environmental regulation will hamper green innovation activities. Barbara and Mconnell [33] selected five heavily polluting industrial sectors in the United States for analysis. They found that environmental regulation generally inhibits green productivity, which is not conducive to green innovation. Greenstone et al. [34] believed that environmental regulation will increase corporate costs and inhibit technological innovation. Montalvo [35] conducted an analysis of variance and correlation analysis of clean technology of enterprises in northern Mexico. They pointed out that ignoring technological trends and too strict environmental regulations would hinder enterprises from green innovation. Based on the data of German manufacturing enterprises, Wagner [36] found that the standard level of environmental regulation implementation has hindered green innovation to some extent. Chintrakarn [37] researched on manufacturing in 48 states of the United States, and they found that the higher the level of environmental regulation, the more positive impact on green technology inefficiency.

The third viewpoint is that there is a U-shaped (inverted U-shaped) relationship between environmental regulation and green innovation. There is an “inflection point” of the intensity of environmental regulation and crossing the inflection point can play a role in promoting (inhibiting) green innovation through environmental regulation. Peng et al. [38] found that there is a U-shape relationship between formal environmental regulation and green innovation efficiency, while there is an inverted U-shaped relationship between informal environmental regulation and green innovation efficiency. Guo et al. [39] examined the effects of environmental regulation and government R&D expenditure for green innovation, and they found that there exists an “inflection point” in the role of environmental regulation in green innovation, and China is at the stage of inhibition before the “inflection point”. 

The fourth viewpoint is that the role of environmental regulation on green innovation is uncertain. In general, the role of environmental regulation in green innovation cannot be summarized simply by inhibition or promotion. The main manifestation is that the impact of environmental regulation on green innovation will vary due to differences in industries, periods, and regions. Frondel et al. [40] pointed out that on the one hand, the command-control environmental regulation policy plays an important role in promoting the development of pollution end-control technology, but its role in integrated cleaner production technology is not obvious; On the other hand, the positive incentive effect of market-incentive environmental regulation policies is not obvious. Wang and Jiang [41] proposed that environmental regulation has an inhibitory effect on green product and process innovation in mining, and it has a significant positive impact on green product and process innovation in the primary processing industry.

#### 2.1.2. The Relationship between Two-Way FDI and Green Innovation

International direct investment (i.e., two-way FDI) is divided into inward foreign direct investment (IFDI) and outward foreign direct investment (OFDI). The relationship between IFDI and green innovation has been thoroughly studied in the existing literature. For instance, Bi et al. [42] conducted an empirical test on the impact of IFDI inflows on the green innovation capability of China’s manufacturing green innovation system. They found that IFDI inflow has a positive effect on the input of various elements of the green innovation resources, but the IFDI information inflow has inhibited the green innovation human resources; and innovation resource input plays a full intermediary role in the process of IFDI inflow affecting the green innovation capability. Song et al. [43] took the China’s regional sample as an empirical research object. They found that IFDI has not only improved China’s investment environment and promoted the rapid growth of China’s economy, but also improved the green innovation capability of Chinese enterprises through technology spillovers.

Scholars have carried out in-depth research on the relationship between OFDI’s reverse technology spillover and technological innovation. Scholars’ opinions differ, and they can be divided into the following three categories. The first category is that OFDI’s reverse technology spillovers can boost the performance of technological innovation in the country. Kogut and Chang [44] verified the existence of reverse technology spillover effects for Japanese investment enterprises in the United States. Coe and Helpman [45] first used the international R&D spillover regression method to empirically derive the existence of R&D spillovers through import trade. Branstetter [46] empirically analyzed the technology spillover effect brought by OFDI between Japan and the United States at the enterprise level, and believed that there was a two-way spillover effect. OFDI can bring advanced experience and technology from the host country and promote technological progress in the home country; that is, there is a reverse technology spillover effect [47,48,49,50].

The second category is that the reverse technology spillover of OFDI has no significant effect on the improvement of domestic technological innovation performance. Btitzer and Görg [51] examined industry-level data of 17 countries in the Organization for Economic Cooperation and Development (OECD) and found that the impact of OFDI on total factor productivity is negative. Lee [52], and Bitzer and Kerekes [53] also reached similar conclusions. For the role of OFDI’s reverse technology spillovers on China’s scientific and technological progress, Li and Liu [54] considered it to be weak, and even a hindrance.

The third category is that the impact of OFDI’s reverse technology spillover on the performance of domestic technological innovation requires a comprehensive analysis, which is a non-linear relationship, and generally introduces threshold variables. Whether OFDI can generate technology spillovers is closely related to the level of development of the host country and region. Only when this threshold is exceeded can a good spillover be produced. This is the threshold effect; Borensztein et al. [55] and Görg and Greenaway [56] found that OFDI can promote technological progress in the home country, and it is related to the absorption capacity of the home country in terms of human capital. They concluded that before human capital reaches the “threshold value”, it can have a significant spillover effect on the technological progress of the home country. 

While paying attention to the relationship between OFDI’s reverse technology spillover and technological innovation, some research has begun to pay attention to the relationship between OFDI and green innovation in China. Song and Du found that in the process of international technology spillover, OFDI reverse technology spillover has a negative impact on green innovation in China [57]. Jia et al. [31] found that China’s OFDI, which invests in developed and developing countries, has promoted the research and development of green technology in the home country. Gong et al. [9] found that OFDI has significantly promoted the efficiency of industrial green innovation through the three mechanisms of agglomeration structure lightening effect, agglomeration economies of scale, and agglomeration resource allocation effect. 

Existing studies mainly focused on the independent analyzing the influence mechanism and role of one specific factor (such as ER, IFDI, and OFDI) on green innovation. However, few literatures have attempted to integrate ER, IFDI, and OFDI in the unified analysis framework to analyze their impact on green innovation. For instance, Li and Zhang [58] analyzed the impact of two-way FDI on green innovation under the conditions of environmental regulation in China, and they found that, under the conditions of environmental regulation, IFDI and OFDI have positive effects on green innovation in the whole country, as well as in the eastern and central regions, while the impact on the western region is not significant.

The above studies provide useful inspiration and references for this article, which carries out the following main expansions: first, the research on international direct investment on green innovation is still in its infancy. The existing research mainly explores the impact of IFDI and OFDI on green innovation based on independent perspectives. This paper attempts to systematically examine the effect of two-way FDI on green innovation in the manufacturing industry under a unified analysis framework and verify the true green innovation effect of IFDI and OFDI. Second, under certain conditions, environmental regulation can affect the innovation spillover of two-way FDI. Existing studies focuses on the relationship between environmental regulation and green innovation. However, there is little literature on whether environmental regulation affects green innovation in China through international direct investment. This paper adds the interactions of environmental regulation and two-way FDI to the research process to empirically verify whether this green innovation spillover path exists? Third, on the basis that China’s manufacturing industry is classified as patent-intensive manufacturing and non-patent-intensive manufacturing, this study measures and analyzes the industry heterogeneity characteristics of two-way FDI affecting green innovation. It provides reference for rational policy formulation of international direct investment.

### 2.2. Research Hypothesis

#### 2.2.1. Environmental Regulation and Green Innovation

Compared with general technological innovation, green innovation has “double externality”, which brings economic benefits to enterprises while also having certain environmental and social benefits [59]. The environmental and social benefits brought by green innovation tend to be higher than the benefits of enterprises, while the enterprises bear the all cost of green innovation, but fail to reap the full innovation revenue. Therefore, due to the lack of government intervention, the incentives for enterprises to carry out green innovation are insufficient, which ultimately leads to serious shortage of green innovation. Eventually, the investment and R&D level of green innovation are long under the social optimal scale. The government applies appropriate incentives and constraints to enterprises through environmental regulation policies, which in turn drives enterprises to carry out green innovation. This is one side. On the other hand, in the context of increasing environmental regulation, enterprises have to increase investment in environmental protection and pollution control in order to meet environmental regulation requirements, thereby increasing the production costs and indirectly reducing the market competitiveness of enterprises. In order to reduce the cost brought by environmental regulation, enterprises will use green innovation to reduce front-end pollution discharge and end treatment investment. Carrying out green innovation is an effective measure for enterprises to cope with environmental regulation, digest and reduce environmental control cost, and tap new growth points of economic benefits [60]. China’s formal institution is not perfect; it needs the self-motivation and self-restraint role of environmental regulation to supplement the formal institution and to strengthen the cooperation between government and enterprises, thus contributing to the green innovation of enterprises. Based on the above analysis, the first hypothesis is stated as follows.

**Hypothesis 1** **(H1).** *Environmental regulation has a positive impact on green innovation.*

#### 2.2.2. Two-Way FDI and Green Innovation

IFDI technology spillovers are the external economic effects of local technology or productivity advancement caused by IFDI. IFDI promotes technology spillovers through the demonstration, competition, flow, and correlation effects. IFDI technology spillovers provide important support for corporate green innovation [42]. From the demonstration effect, IFDI technology spillovers not only bring new equipment, new products and new technologies to local enterprises, but also bring advanced management experience to the host country. Local enterprises enhance the level of green innovation on the basis of external knowledge acquisition and internalization. From the perspective of the competition effect, after IFDI enters the host country, market competition intensifies. Local enterprises pay more and more attention to investment in learning. The greater the investment in learning, the higher the technical and knowledge base of local enterprises, which is conducive to the development of green innovation activities. From the flow effect, IFDI spillovers promote the growth of human capital in local enterprises through the flow of personnel to realize the absorption and transformation of external knowledge and accelerate the process of green innovation of enterprises. From the correlation effect, foreign enterprises put forward higher requirements for product quality in the upstream and downstream of local enterprises, so local enterprises increase relevant investment and improve technology levels, quality standards, and the green innovation process. Based on the above analysis, the research hypothesis 2 is proposed:

**Hypothesis 2** **(H2).** *IFDI has a positive impact on green innovation.*

OFDI is an act of capital export by investors to open up the international market. It links the domestic and international markets, which promotes the rational circulation and allocation of resources. The key to green innovation is to continuously increase investment in innovative resources. Therefore, in the process of OFDI, Chinese enterprises can bring certain green innovation resources to the home country in different ways and improve the level of green innovation in China [61]. Enterprises are exposed to more advanced green production technologies in the international market through OFDI. In order to improve their level of green innovation, enterprises will acquire advanced foreign technologies through purchases, mergers and acquisitions and so on. Enterprises generate knowledge accumulation in the process of technology application, and finally “spill” this knowledge into the home country, resulting in reverse green technology spillover effects. In this way, the level of green innovation in the home country will be improved, so that enterprises can better consolidate the domestic market share. In addition, in the process of OFDI, enterprises learn from advanced foreign green technologies and draw on the experience of leading foreign green production management. Enterprises then apply these technologies and experiences to domestic green innovation, and they promote the transformation of extensive production modes in order to adapt to domestic and international environmental regulation standards, and improve the domestic and international competitiveness of products. Based on the above analysis, the following research hypothesis can be proposed:

**Hypothesis 3** **(H3).** *OFDI has a positive impact on green innovation.*

#### 2.2.3. The Regulatory Role of Environmental Regulation between Two-Way FDI and Green Innovation

In the early stage of reform and opening up, weaker environmental regulation intensity and a less perfect environmental governance system led to the transfer of a large number of polluting technologies and industries to China, which led to IFDI primarily investing in pollution-intensive industries. With the strengthening of environmental regulation, all regions are paying more attention to the introduction of high-quality funds, especially those with relatively high technology content, when attracting IFDI, which promotes the green innovation performance of enterprises. Environmental regulation policy is an important threshold for foreign investment, technology introduction, and industrial transfer, and it imposes additional environmental governance costs on foreign-funded enterprises, especially foreign-funded enterprises that pollute. This, to a certain extent, prevents the entry of polluting foreign capital and encourages the entry of clean production-oriented foreign capital, which can promote the transformation of green innovation development in China’s manufacturing industry. Therefore, the appropriate implementation of environmental regulation policy is a compulsory “fine wash” for IFDI in China, and it has the function of “decontamination and clearing” [62,63], which can guide IFDI to promote green innovation in manufacturing. In other words, the interaction between environmental regulation and IFDI may promote green innovation in manufacturing. Based on the above analysis, the research hypothesis can be proposed:

**Hypothesis 4** **(H4).** *Environmental regulation positively regulates the impact of IFDI on green innovation.*

In order to encourage polluting enterprises to adopt advanced green technologies to improve or change production processes and reduce environmental pollution, the government has adopted strict environmental regulations to increase the production costs of sewage enterprises. Moreover, enterprises with advanced green technologies can offset, or partially offset the increased costs of responding to environmental regulations by effectively expanding market share, and they can obtain innovative compensation induced by environmental regulations. Due to the externalities of green innovation, many domestic enterprises have been inclined to develop and utilize non-green technologies, and there is no incentive for the development and application of green technologies. Under the influence of China’s environmental regulation, enterprises may use OFDI to developed countries to obtain advanced green technologies from host countries, and then spill these effects over into green innovation in China. Studies have shown that China’s technology-seeking OFDI has accounted for more than 20% of total foreign investment, so the reverse green technology spillover through OFDI is an important channel for international green technology diffusion [64]. Based on the above analysis, the research hypothesis can be proposed:

**Hypothesis 5** **(H5).** *Environmental regulation positively regulates the impact of OFDI on green innovation.*

**Figure 1:**
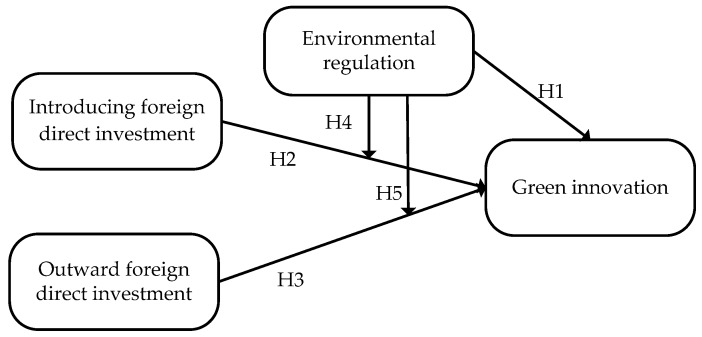
The theoretical model of hypotheses.

In summary, the theoretical model of the hypotheses constructed in this paper is shown in Figure 1.

## 3. Empirical Design

### 3.1. Model Design

The factors affecting green innovation are multi-faceted; these include domestic and international factors. This paper primarily analyzes the impact of two-way FDI on green innovation under environmental regulation conditions from the perspective of international capital flows. In order to systematically examine the effects of environmental regulation and two-way FDI on the green innovation efficiency of the manufacturing industry, the following measurement model is constructed:

(1) $$GIE_{it}=C+\beta_{1}ER_{it}+\beta_{2}IFDI_{it}^{}+\beta_{3}OFDI_{it}^{}+\beta_{4}CI_{it}+\beta_{5}SE_{it}+\beta_{6}GS_{it}+\beta_{7}PRS_{it}+\varepsilon_{it}$$

In this Model (1), *i* represents the industry and *t* represents the period; *C* represents a constant term; *ε* represents the random error term. In addition, *GIE_it_* represents green innovation efficiency of manufacturing industry, *ER_it_* represents environmental regulation, *IFDI_it_* represents inward foreign direct investment, *OFDI_it_* represents outward foreign direct investment, *SE_it_* represents scale of enterprise, *CI_it_* represents capital intensity, *GS_it_* represents government support, and *PRS_it_* represents property right structure. 

Environmental regulation may affect the green innovation efficiency of the manufacturing industry by influencing IFDI and OFDI, which means that environmental regulation, has a regulatory effect on the impact of two-way FDI on green innovation efficiency. Thus, this paper introduces the interactions between environmental regulation and two-way FDI. Here is Model (2):

(2) $$\begin{array}{ll} GIE_{it} & =C+\beta_{1}ER_{it}+\beta_{2}IFDI_{it}^{}+\beta_{3}OFDI_{it}^{}+\beta_{4}CI_{it}+\beta_{5}SE_{it}+\beta_{6}GS_{it}+\beta_{7}PRS_{it}+\beta_{8}ER_{it}*IFDI_{it}^{}\\ & +\beta_{9}ER_{it}*OFDI_{it}^{}+\varepsilon_{it} \end{array}$$

### 3.2. Selection of Variables

#### 3.2.1. Explained Variable: Green Innovation Efficiency

(1) Method for measuring green innovation efficiency

It is generally known that the model structure is one of the major factors influencing the model performance [65,66]. Traditional CCR and BCC models of data envelopment analysis (DEA) measure efficiency from both radial (i.e., ratio of input and outpu) and angular (i.e., input or output angle) perspectives. Because the slack problem of input and output is not considered, the accuracy of efficiency measurement is affected. Therefore, Tone proposed a non-radial, non-angled SBM model based on the measure of slack variables [67]. However, the SBM model cannot further evaluate and rank effective decision-making units with an efficiency value of 1. Tone further proposed a super-efficient SBM model (i.e., the Super-SBM model) that introduces undesired outputs [68]. Suppose a production system has *n* decision units (DMU), and each DMU contains three factor vectors: input, desired output, and undesired output; they are $x\in R^{m}$, $y^{d}\in R^{s_{1}}$, $y^{u}\in R^{s_{2}}$, where *m, s*_1_ *and s*_2_ represent the kind of input, desired output, and undesired output, respectively. The corresponding matrix is *X, Y^d^, Y^u^*, and can be expressed as: *X* = [*x*_1_, …, *x_n_*] ∈ *R^m^*^×*n*^ > 0, *Y^d^* = $\left[y_{1}^{d},\ldots ,y_{n}^{d}\right]\in R^{s_{1}\times n}>0$, *Y^u^* = $\left[y_{1}^{u},\ldots ,y_{n}^{u}\right]\in R^{s_{2}\times n}>0$, the SBM model with undesired output can be expressed as:

(3) $$\begin{array}{c} \min\rho =\frac{1-\frac{1}{m}\sum {}_{i=1}^{m}s_{i}^{-}/x_{i0}}{1+\frac{1}{s_{1}+s_{2}}(\sum {}_{r=1}^{s_{1}}\frac{s_{r}^{d}}{y_{r0}^{d}}+\sum {}_{r=1}^{s_{2}}\frac{s_{l}^{u}}{y_{l0}^{d}})}\\ s.t.\;:x_{i0}=\sum_{j=1}^{n}x_{ij}\lambda_{j}+s_{i}^{-},\;i=1,\;\cdot \cdot \cdot ,\;m;\;y_{r0}^{d}=\sum_{j=1}^{n}y_{j}^{d}{}_{r}\lambda_{j}-s_{r}^{d},\;i=1,\;\cdot \cdot \cdot ,\;s_{1}\\ y_{r0}^{u}=\sum_{j=1}^{n}y_{ij}^{u}\lambda_{j}+s_{i}^{u},\;l=1,\;\cdot \cdot \cdot ,\;s_{2};\;\lambda_{j}>0,\;\sum \lambda_{j}=1,\;s_{i}^{-}\geq 0,\;s_{r}^{d}\geq 0,,\;s_{l}^{u}\geq 0 \end{array}$$

In Equation (3), ρ is the target efficiency, and 0≤ρ≤1; $s_{}^{-}$, $s_{}^{d}$, and $s_{}^{u}$ represent relaxation variables of factor inputs, desired outputs, and undesired outputs; λ is the weight vector, subscript “0” indicates the evaluated decision unit. If, and only if, ρ=1 and $s_{}^{-}=0$, $s_{}^{d}=0$, and $s_{}^{u}=0$, the evaluated decision unit is valid; ρ<1 indicates that the evaluated decision unit is invalid, and the input and output need to be adjusted.

Considering that the SBM model with the undesired output of the decision-making unit will be effective in the meantime (i.e., the efficiency values are all 1), it is not conducive to evaluation decision-making unit. Therefore, the Super-SBM model should be used, so that efficiency values above 1 are allowed. The reference introduces undesired outputs into the SBM model, which can be introduced into the Super-SBM model:

(4) $$\begin{array}{c} \min\delta =\frac{\frac{1}{m}\sum {}_{i=1}^{m}\bar{x}/x_{i0}}{\frac{1}{s_{1}+s_{2}}(\sum {}_{r=1}^{s_{1}}\frac{{\bar{y}}^{d}}{y_{r0}^{d}}+\sum {}_{r=1}^{s_{2}}\frac{{\bar{y}}^{u}}{y_{l0}^{u}})}\\ s.t.\;:\bar{x}\geq \sum_{j=1,\neq 0}^{n}y_{lj}^{u}\lambda_{j},\;i=1,\;\cdot \cdot \cdot ,\;m;\;{\bar{y}}_{}^{d}\leq \sum_{j=1,\neq 0}^{n}y_{j}^{d}{}_{r}\lambda_{j},\;r=1,\;\cdot \cdot \cdot ,\;s_{1}\\ {\bar{y}}_{}^{u}\geq \sum_{j=1,\neq 0}^{n}y_{ij}^{u}\lambda_{j},\;l=1,\;\cdot \cdot \cdot ,\;s_{2};\;\bar{x},\geq x_{ij}\;{\bar{y}}^{d}\leq y_{rj}^{d},\;{\bar{y}}^{u}\geq y_{lj}^{u},,\;\lambda_{j}\geq 0 \end{array}$$

The Super-SBM model can effectively solve the problems of undesired output, slackness, and multiple DMUs being effective at the same time in efficiency evaluation.

(2) Measuring indicators of green innovation efficiency

Input indicator: drawing on existing research results, the green innovation investment in the manufacturing industry includes three main aspects: human input, capital investment, and energy input. Among them, R&D active staff full-time equivalent as a measure of human resource input for manufacturing green innovation activities. In addition, besides intellectual support, the green innovation process requires material resources or material capital to provide support and preconditions. The intramural expenditures on R&D is a key indicator for measuring the material resources required in the green innovation process, it is mainly used for the consumption of mobile capital and the increase of fixed capital, and the intramural expenditures on R&D is used as a measure of the input of material resources for green innovation activities in manufacturing. Considering that the research object of this article is the green innovation efficiency, in order to better explore the impact of resource consumption on innovation efficiency, the indicators that can reflect energy consumption are supplemented on the basis of the above non-resource elements. It is proposed that the energy consumption of the energy terminal be selected; that is, the total energy consumption of each industry was selected as the energy input factor. 

Output indicator: including both expected and undesired outputs. On the measurement of desired output indicators for green innovation in manufacturing industry, two consecutive processes of knowledge and product production for green innovation activities should be considered. The domestic inventive patent applications granted was selected as an output variable to reflect the knowledge output level of the green innovation process. However, inventive patents cannot accurately reflect the transformation ability and market value of innovation results, as there are significant limitations in measuring the economic benefits of green innovation. The new product sales revenue indicator can effectively measure the market value of innovation results, and it represents the new products or services that the company will develop. The economic benefits that they can bring to a company after entering the market are the quantification of the quality of innovations and the reasonable response to the commercialization of innovations. Therefore, the sales revenue of new products is used to measure the economic benefits brought by green innovation. Under the constraints of the energy environment, the manufacturing industry begins green innovation activities, and the effect is to generate three types of benefits: economic, environmental, and social. Nowadays, the environmental and social benefits that are brought through the implementation of green innovation in the manufacturing industry should be reflected mainly in their contribution to reducing environmental pollution and creating an environment-friendly society. The environmental and social benefits of green innovation in manufacturing are primarily reflected in the reduction of environmental pollution; this means less undesired output would be better. Regarding the selection of undesired outputs, the current research has not yet reached a consensus. If only one type of pollutant is selected, it is an undesired output indicator, which will cause the research results to not reflect the real situation. This study selects industrial wastewater discharge, industrial waste gas emissions, and industrial solid waste production in the manufacturing subsectors as the undesired output to measure the environmental benefits brought by green innovation in the manufacturing industry.

#### 3.2.2. Core Explanatory Variables

The core explanatory variables of this article are environmental regulation, IFDI, and OFDI.

Environmental regulation: The famous “Porter Hypothesis” was officially proposed in the 1990s. The “Porter Hypothesis” suggests that reasonable environmental regulation has the effect of innovative compensation, which is the appropriate environmental regulation, can compensate for the cost of the regulation and promote innovation. On the basis of the “Porter Hypothesis”, environmental regulation has been used by domestic and foreign scholars as an important factor influencing green innovation and promoting green transformation. For environmental regulation, there is currently no unified measurement form, and investment in industrial pollution control is a common indicator. However, in view of the fact that the public statistics do not have pollution-related investment data, referring to Gray [69], and Seiford and Zhu [70], this study uses pollution facility operating costs instead of pollution control investment, and calculates the sum of the operating costs of industrial wastewater treatment facilities and the operating costs of industrial waste gas treatment facilities, which is divided by the total output value as the measure indicator of environmental regulation.

As for IFDI and OFDI, current published statistics only report major industry categories of IFDI and OFDI, the segmentation of IFDI and OFDI in the double-digit industrial sector has not been reported, and there is a significant export-creating effect of China’s OFDI on major countries. Therefore, this paper uses multiply total IFDI and OFDI by the proportion of each manufacturing export to approximate representation the IFDI and OFDI flow data of the manufacturing subsectors [71]. On this basis, the ratio of IFDI and OFDI flow data to the total industrial output value is used to measure IFDI and OFDI in each industry.

#### 3.2.3. Control Variables

According to the empirical research literature in the field of innovation, in order to obtain unbiased estimation results, the control variables of this paper are selected as follows:

Capital intensity: In general, if the rate of capital increase is faster than the increase in labor, the green innovation efficiency in the industry would be higher. Industrial capital intensity is expressed by dividing the industry’s net fixed assets by the employee number.

Scale of enterprise: In a mature economy, large enterprises bear a share of innovation beyond their proportion, and there is a relationship that cannot be ignored between scale of enterprise and innovation efficiency. For enterprises, the costs and risks of green innovation are high. It is generally believed that large enterprises are rich in funds and strong in research and development, and they have certain technical preferences for the choice of innovation strategies. Large enterprises often choose green innovation, which is a forward-looking major technological innovation. Therefore, the scale of enterprise may have a positive impact on the company’s initiative to carry out green innovation. The ratio of the total industrial output value to the number of industrial enterprises is used to represent the scale of enterprise.

Governmental support: Innovation activities have a certain “externality”, which characterizes an enterprise’s green innovation activities as high cost and high risk. The government compensates for part of the cost of green innovation through direct R&D investment, which can reduce the risk of green innovation, but this behavior may bring a “crowding-out effect” to corporate green innovation and reduce innovation performance. Government support is measured by the proportion of government funds in the intramural expenditures on R&D.

Property right structure: For countries such as China where the market economy is still immature, the property right structure is a special factor affecting the efficiency of innovation [72]. This indicator is measured by the proportion of state-owned and state-controlled enterprises in industrial output value.

### 3.3. Data Source and Description

#### 3.3.1. Data Sources

According to the classification of the national industrial economy promulgated by China’s National Bureau of Statistics, the manufacturing industry includes all industries except the extractive industry and public utilities, with a total of 29 industries. Since 2012, the scope of statistics in the relevant statistical yearbooks has changed. Therefore, based on the consistency of industry classification and the availability of data, this article combines the rubber products industry and the plastic products industry into rubber and plastic products industry, and automobile manufacturing industry and railway, shipbuilding, aerospace and other transportation equipment manufacturing industry merge into manufacture of transport equipment, and deletes crafts and other manufacturing or other manufacturing industries; a total of 27 manufacturing industries are selected. Since the “China science and technology statistical yearbook 2010” began to count “R&D activities staff and expenditure”, the previous statistics were “scientific and technological activities staff and expenditure”, resulting in “R&D active staff full-time equivalent” and “the intramural expenditures on R&D”, which only became available in 2009. Therefore, this article takes panel data for 27 industries in China’s manufacturing industry from 2009 to 2015 as a sample.

#### 3.3.2. Data Adjustment

(a) The price-related indicator data is deflated. In order to eliminate the impact of price changes, the actual value of the base period (i.e., the year 2009) is calculated for all variable indicators involving prices using the industrial producer ex-factory price index of each industry over the years.

(b) The cumulative nature of R&D expenditure. The intramural expenditures on R&D reflects the actual innovation capital investment of the executing units during the year and is a flow indicator. However, the impact of innovation activities on knowledge production is not only reflected in the current period, but also has an impact on future knowledge production. Therefore, the intramural expenditures on R&D flow data should be replaced by the intramural expenditures on R&D stock. Using the perpetual inventory method to estimate the intramural expenditures on R&Ds stock in the manufacturing subsectors [73], the calculation formula is:

(5) $$k_{t}=(1-\delta )k_{t-1}+E_{t-1}$$

In Equation (5), $k_{t}$ and $k_{t-1}$ respectively represent the intramural expenditures on R&D stock of the t and t−1 periods of each industry, and $E_{t-1}$ represents the actual intramural expenditures on R&D in the t−1 period of each industry (i.e., the intramural expenditures on R&D adjusted according to the industrial producer’s ex-factory price index). *δ* is the depreciation rate; *δ* = 15%. The intramural expenditures on R&D stock in the base period (i.e., the year 2009) is based on the steady state method by Harberger [74], and it is estimated by the formula of $k_{0}=E_{0}/(g+\delta )$. Among them, g is the arithmetic average growth rate of intramural expenditures on R&D in various industries from 2009 to 2015.

The research data for this article comes mainly from the “China Science and Technology Statistical Yearbook”, “China Industrial Economics Statistical Yearbook”, “China Foreign Direct Investment Statistics Bulletin”, “China Statistical Yearbook”, and “China Environmental Statistical Yearbook”, and the statistical caliber are “industrial enterprises above designated size.” Details of the variables are outlined in Table 1.

## 4. Empirical Results and Analysis

### 4.1. The Result of Measuring the Green Innovation Efficiency of the Manufacturing Industry

This study uses MaxDEA Pro software to calculate the green innovation efficiency of China’s 27 manufacturing industries from 2009 to 2015 based on the Super-SBM model. The calculation results are shown in Table 2. The results show that the mean of green innovation efficiency of China’s manufacturing industry from 2009 to 2015 is 0.4697. Among them, 16 industries, such as Manufacture of beverages, have a mean of green innovation efficiency that is lower than the mean of whole manufacturing industry. Overall, the green innovation efficiency of China’s manufacturing industry is low, and there is room for improvement.

In the period 2009–2015, in terms of industries, the five industries with the highest mean of green innovation efficiency are: manufacture of furniture (1.0942), manufacture of articles for culture, education and sport activity (1.0351), manufacture of communication equipment, computers and other electronic equipment (0.9855), manufacture of electrical machinery and equipment (0.9541), and manufacture of tobacco (0.9234). The means of green innovation efficiency of these five industries are all greater than 0.9. The four industries with the lowest mean of green innovation efficiency are: manufacture of beverages (0.1200), manufacture of paper and paper products (0.1356), manufacture of chemical fibers (0.1733), and processing of petroleum, coking, processing of nuclear fuel (0.1902), and the means of green innovation efficiency of these four industries are all less than 0.2.

In order to further compare the industry differences in green innovation efficiency of China’s manufacturing industry, this article classifies and analyzes 27 manufacturing industries as patent-intensive or non-patent-intensive manufacturing. The patent-intensive industry is a collection of enterprises with key technologies and core patents. It has a significant impact on implementing innovation-driven development strategies, realizing industrial transformation and upgrading, and promoting sustained economic growth.

The Patent Intensive Industry Catalogue (2016) issued by the State Intellectual Property Office of China (Trial) has identified 48 national economy industries as patent-intensive industries. This catalogue corresponds to the middle category (three-digit code) of the national economic industry classification (GB/T 4754-2011). According to the two-digit code manufacturing industry of 48 three-digit code industries belong to, this paper finalizes the following 10 manufacturing industries as patent-intensive manufacturing: raw chemical materials and chemical products; manufacture of medicines; smelting and pressing of non-ferrous metals; manufacture of metal products; manufacture of general purpose machinery; manufacture of special purpose machinery; manufacture of transport equipment; manufacture of electrical machinery and equipment; manufacture of communication equipment, computers and other electronic equipment; manufacture of measuring instruments and machinery for cultural activity and office work. The rest are non-patent-intensive manufacturing. From the perspective of green innovation efficiency, the mean of green innovation efficiency of patent-intensive manufacturing is 0.5852, and the mean of green innovation efficiency of non-patent-intensive manufacturing is 0.4018; this result further reveals the industry heterogeneity of green innovation efficiency in the manufacturing industries, which is consistent with each industry’s own characteristics and technical features.

### 4.2. Measurement Test Results and Analysis

On the basis of the basic Models (1) and (2), the 27 manufacturing industries, 10 patent-intensive manufacturing industries, and 17 non-patent-intensive manufacturing industries, are used to analyze the effects of environmental regulation and two-way FDI on the green innovation efficiency of China’s manufacturing industry. Using Eviews software, the fixed effect model or the random effect model was selected by the Hausman test to regress the sample data. When the Hausman test results were significant at the 10% level, the fixed effect model was selected; otherwise the random effect model was selected. The estimation results are shown in Table 3. Based on the model estimation results, Table 4 shows the comparisons of research hypothesis and empirical results.

#### 4.2.1. The Influence of Environmental Regulation on the Green Innovation Efficiency of China’s Manufacturing Industry

For Model (1), in manufacturing, patent-intensive manufacturing, and non-patent-intensive manufacturing, the impact of environmental regulation (ER) on green innovation efficiency is significantly negative. A possible explanation is that the measurement index in this paper is the cost of enterprises to deal with environmental regulation (i.e., the environmental investment and pollution control investment of enterprises). For the economic entity of Chinese manufacturing enterprises, the cost of responding to environmental regulation will increase their production cost, but this expense is relatively low in their total cost. Therefore, the enterprises’ rational choice of productive investment (e.g., adjustment of the original production decision or industrial structure) can make up for the cost brought by environmental regulation, and then ignore the pollution-control effect of green innovation. Enterprises are more willing to undertake environmental regulation than through green innovation to reduce pollutant emissions, and even some enterprises use the funds originally used for green innovation to bear the cost of environmental regulation in order to obtain short-term higher financial goal. That is to say, the “crowding-out effect” caused by environmental regulation makes the reduction of enterprises’ green innovation investment, and thus has a restraining effect on green innovation. At present, the intensity of environmental regulation of China’s manufacturing industry is relatively low, and the “follow the cost effect” caused by environmental regulation is greater than the “compensation effect” of green innovation, which in turn reduces the green innovation efficiency.

#### 4.2.2. The Influence of Two-Way FDI on the Green Innovation Efficiency of China’s Manufacturing Industry

For Model (1), in patent-intensive manufacturing and non-patent-intensive manufacturing, IFDI has a significant positive impact on green innovation efficiency. The Chinese government is paying more and more attention to the entry structure for IFDI and strengthening the approval of pollution-based IFDI projects. The advanced production and pollution control technologies brought about by the inflow of IFDI not only effectively reduce their own pollution emissions, but also drive local enterprises to green production through competition, demonstration, and learning effects as well as promote the improvement of green innovation efficiency and green technology progress in the manufacturing industry. Due to the high cost of green innovation funds in patent-intensive manufacturing, it is necessary to continuously rely on the spillover effect of foreign investment. Multinational enterprises in China’s R&D institutions gradually carry out research and development on the green and clean production technology, these R&D activities and their technology spillover effects have driven the frontier of green technologies in China’s patent-intensive manufacturing industry, and they have improved the green innovation efficiency. 

In manufacturing, patent-intensive manufacturing, and non-patent-intensive manufacturing, OFDI has a positive impact on green innovation efficiency, and passes the significance test in manufacturing, which shows that OFDI has a certain positive impact on the improvement of the green innovation efficiency of China’s manufacturing industry. A possible explanation is that OFDI of China’s manufacturing industry has a significant reverse innovation spillover effect. Through OFDI, enterprises can access more advanced green technologies in the process of communication with enterprises and research institutions in host country. In order to adapt to the environmental regulation standards in host country, enterprises generally take the initiative to improve their own level of green production, and acquire advanced foreign green technologies through purchase and mergers and acquisitions. At the same time, enterprises will reverse the advanced green knowledge and technologies to the home country, and promote the efficiency of domestic green innovation through effective diffusion, demonstration, absorption, and secondary innovation, which is the reverse green technology spillover effect.

#### 4.2.3. Test of Control Variables

For capital intensity (CI), its coefficient is not significantly negative in the patent-intensive manufacturing and non-patent-intensive manufacturing; in the manufacturing industry, the coefficient is not significantly positive. In recent years, the development trend of China’s heavy industrialization is obvious, and heavy industrialization has led to the deterioration of environmental quality. The coefficient of scale of enterprise (SE) is significantly positive; indicating that the increase in the proportion of large and medium-sized enterprises in the industry has a positive impact on the green innovation efficiency of China’s manufacturing industry. The coefficient of government support (GS) is negative, and it passes the significance test in non-patent-intensive manufacturing; it indicating that Chinese government’s R&D support for the enterprises failed to achieve its original purpose, and failed to effectively promote industrial green innovation. The regression coefficient of the property right structure (PRS) is negative, but not significant, indicating that the state-owned property rights and the green innovation efficiency are negatively related; the reason is that the lack of incentives for state-owned property rights has a strong inhibitory effect on high-risk behaviors such as green innovation.

#### 4.2.4. The Influence of the Interaction between Environmental Regulation and Two-Way FDI on the Green Innovation Efficiency of China’s Manufacturing Industry

For Model (2), in manufacturing and non-patent-intensive manufacturing, the impact of interaction items *ER × IFDI* on green innovation efficiency is positive, and it passes the significance test in non-patent-intensive manufacturing. When there are no environmental regulations or low levels of environmental regulations in China, developed countries with more stringent environmental regulations will transfer their polluting industries to China. However, with the emphasis on environmental pollution in China, it has begun to develop more complete and strict environmental regulations. China raises the environmental threshold for foreign investment and plays a “screening” role for IFDI, giving priority to IFDI that is conducive to environmental protection and technological upgrading. Meanwhile, the stricter environmental regulation will also promote the proportion of research and development funds in IFDI, and gradually develop green and clean production technology.

For Model (2), the interaction items *ER ×* *OFDI* has a negative impact on green innovation efficiency, and it passes the significance test in manufacturing and patent-intensive manufacturing, and the coefficients are respectively −0.0468 and −0.0697. A possible explanation is that OFDI has a self-selective effect, and this effect will be more pronounced under environmental regulations. On the one hand, for manufacturing enterprises, especially non-patent-intensive manufacturing enterprises with low levels of green development, in the face of increasingly stringent domestic environmental regulation measures, they will transfer the production of some pollution-intensive industries or businesses abroad, thereby reducing the cost of coping with environmental regulation. Therefore, the OFDI of China’s manufacturing industry may have a motive for pollution transfer. On the other hand, due to difficulties in overseas financing, most of the current Chinese enterprises’ OFDI funds come from inside the company. At this time, OFDI of enterprises may reduce their funds in China and tighten their domestic liquidity constraints, including the need for R&D capital investment in innovation, which hinders enterprises from promoting green innovation. Overall, the test results of other variables in Model (2) are basically consistent with the test results of Model (1).

## 5. Conclusions and Policy Implications

Based on panel data of 27 manufacturing industries in China from 2009 to 2015, this article uses the Super-SBM model to measure the green innovation efficiency of China’s manufacturing industry, and then empirically analyzes the impact of environmental regulation and two-way FDI on the green innovation efficiency of China’s manufacturing industry. The main research conclusions and policy implications are as follows:

First, between 2009 and 2015, the mean of green innovation efficiency in China’s manufacturing industry is 0.4697. Among them, there are 16 industries in which the mean of green innovation efficiency is lower than the mean of the whole manufacturing. Considering the industry heterogeneity, the green innovation efficiency of the patent-intensive manufacturing (the mean is 0.5852) is significantly higher than the green innovation efficiency of the non-patent-intensive manufacturing (the mean is 0.4018). Under the existing technical conditions and resources, there is still much room for improvement in the green innovation efficiency of China’s manufacturing industry. Therefore, at this stage, we should improve the green innovation efficiency by optimizing organizational structure, strengthening internal management, improving management levels, and clarifying the economic and environmental responsibility of the main body of the enterprise, so as to maximize the potential of innovation, and making the boundary of the best production possibilities of green innovation approach the frontier of technology.

Second, the impact of environmental regulation on the green innovation efficiency of the China’s manufacturing industry is significantly negative. The main reason is that the “crowding-out effect” caused by environmental regulation has reduced the investment in green innovation of enterprise. Therefore, it is necessary for the government to formulate reasonable environmental regulation means while enhancing the environmental regulation level of China’s manufacturing industry, and provide a suitable external environment for environmental regulation to stimulate green innovation of enterprise, and motivate it to actively promote green innovation.

Moreover, in the manufacturing, patent-intensive manufacturing, and non-patent-intensive manufacturing, IFDI has a positive impact on green innovation efficiency, and in patent-intensive manufacturing and non-patent-intensive manufacturing has it passed the significance test. In the manufacturing, patent-intensive manufacturing, and non-patent-intensive manufacturing, the impact of OFDI on green innovation efficiency is positive, and in the manufacturing has it passed the significance test. As for this, first of all, the dual role of China as a host country and an investing country in the international capital arena must be fully understood, in order to improve the level of opening up to the outside world, and promote the mode of international direct investment from “tolerant entry and strict exit” to “restricted in and draw forth” transformation. This will actively guide the rational layout of two-way FDI flows, and effectively exert the green technology spillover effect of two-way FDI. For IFDI, we should accelerate the transformation of the previous investment model based on demographic dividend and resource endowment advantages, and vigorously cultivate professional markets to introduce high-tech green foreign investment. For OFDI, we should actively participate in or create a multilateral investment framework to provide comprehensive protection and support for Chinese enterprises’ OFDI in the context of a “going out” strategy; optimize the OFDI structure and improve the overall level of Chinese OFDI; and focus on supporting the increase in technology acquisition investment ratio of the R&D-intensive industries and green industries in developed countries such as Europe and the United States, to achieve an improvement in OFDI “quality” and to maximize the effect of OFDI reverse innovation spillovers. What is more, full play should be given to the guiding role of the government and timely adjustment of relevant policies and measures. The government should actively guide the rational layout of two-way FDI, which should work hard to achieve a coordination between IFDI and OFDI in various industries, and strive to achieve the strengthening effect of “1 + 1 > 2” of two-way FDI on green innovation.

In addition, in manufacturing and non-patent-intensive manufacturing, the impact of interaction between environmental regulation and IFDI on green innovation efficiency is positive, and has passed significant test in non-patent-intensive manufacturing industry. The interaction between environmental regulation and OFDI has a negative impact on the green innovation efficiency, and it passes the significance test in the manufacturing and non-patent-intensive manufacturing. Therefore, while continuing to deepen the concept of green development, the government should fully consider the requirements of environmental protection and the heterogeneity of different manufacturing industries. It should then formulate appropriate levels of environmental regulation according to local conditions and strive to absorb the green innovation spillover of two-way FDI. Meanwhile, it is necessary to dynamically adjust the intensity of environmental regulation in a timely manner and implement flexible, diverse, targeted environmental regulation tools and policies.

Although this study provides valuable insights, it also has limitations. These will be important directions for further research. The shortcomings and research directions are: first, this study is limited to sample data. It can be said that the sample data not only meets the needs of this research, but also represents the typical data for the key period of China’s manufacturing industry green transformation and upgrading. Future research can make progress on data expansion. Second, this study uses a single indicator to measure the environmental regulation level of manufacturing industry, and has not yet classified according to the degree of mandatory environmental regulation. Subsequent research can further classify the impact of environmental regulation on green innovation in manufacturing industry.

## Figures and Tables

**Table 1:** Descriptive statistics of variables.

Variable Type	Variable	Mean	S.D.	Min	Max	Explanation
Explained variable	*GIE*	0.4697	0.3445	0.1153	1.2864	Green innovation efficiency: calculated by Super-SBM
Core explanatory variables	*ER*	0.1327	0.1698	0.0023	0.7999	Industrial wastewater treatment facilities operating costs + industrial waste gas treatment facilities operating costs/total industrial output value (%)
	*IFDI*	0.0609	0.0697	0.0010	0.5404	IFDI flow/total industrial output value (%)
	*OFDI*	0.3997	0.4545	0.0085	2.7448	OFDI flow/total industrial output value (%)
Control variables	*CI*	23.5105	18.1091	3.1964	102.4353	Industrial fixed assets net/employee number (10^4^ yuan/person)
	*SE*	4.7135	10.4729	0.4398	70.2315	Total industrial output value/number of industrial enterprises (100 million/enterprise)
	*GS*	3.1225	2.0677	0.1141	10.4539	The proportion of government funds in the intramural expenditures on R&D (%)
	*PRS*	16.8712	22.1472	0.3897	99.3666	The proportion of state-owned and state-controlled enterprises in total industrial output value (%)

**Table 2:** Evaluation results of green innovation efficiency of China’s manufacturing industry.

Code	Sector	2009	2010	2011	2012	2013	2014	2015	Mean	Rank
C13	Processing of food from agricultural products	0.1559	0.1828	0.1922	0.2441	0.2438	0.2461	0.2559	0.2173	22
C14	Manufacture of foods	0.2130	0.2370	0.1997	0.2157	0.2252	0.2667	0.2398	0.2282	20
C15	Manufacture of beverages	0.1181	0.1267	0.1153	0.1290	0.1153	0.1167	0.1191	0.1200	27
C16	Manufacture of tobacco	0.5666	0.6548	1.1232	1.0003	1.0194	1.0144	1.0848	0.9234	5
C17	Manufacture of textile	0.1392	0.2099	0.2123	0.2196	0.2500	0.2600	0.2900	0.2259	21
C18	Manufacture of textile wearing apparel, foot ware and caps	0.3157	0.4368	0.2629	1.0373	1.0141	0.7410	0.5795	0.6267	8
C19	Manufacture of leather, fur, feather and related products	0.4115	0.4311	0.3199	0.3211	0.2998	0.2635	0.2537	0.3286	13
C20	Processing of timber, manufacture of wood, bamboo, rattan, palm, and straw products	0.3976	0.3470	0.3143	0.2518	0.2241	0.1913	0.1754	0.2717	14
C21	Manufacture of furniture	1.2305	1.1493	1.2125	1.0588	1.0979	0.8524	1.0582	1.0942	1
C22	Manufacture of paper and paper products	0.1335	0.1404	0.1276	0.1301	0.1282	0.1514	0.1382	0.1356	26
C23	Printing, reproduction of recording media	0.5495	0.5762	0.4576	0.5050	0.6358	0.4987	0.4215	0.5206	10
C24	Manufacture of articles for culture, education and sport activity	1.2168	1.2864	0.7023	1.0503	1.0406	1.0981	0.8510	1.0351	2
C25	Processing of petroleum, coking, processing of nuclear fuel	0.1285	0.1167	0.1154	0.1330	0.2571	0.2800	0.3008	0.1902	24
C26	Manufacture of raw chemical materials and chemical products	0.2173	0.2067	0.2425	0.2751	0.2872	0.2884	0.2795	0.2567	17
C27	Manufacture of medicines	0.3581	0.4043	0.3529	0.3989	0.4110	0.4190	0.3188	0.3804	12
C28	Manufacture of chemical fibers	0.1500	0.1438	0.1573	0.1792	0.1738	0.1843	0.2250	0.1733	25
C29	Rubber and plastic products industry	0.2251	0.2442	0.3671	0.2374	0.2761	0.2762	0.2673	0.2705	15
C31	Manufacture of non-metallic mineral products	0.3227	0.1930	0.2210	0.2207	0.2337	0.2297	0.1963	0.2310	19
C32	Smelting and pressing of ferrous metals	0.2180	0.2114	0.2612	0.2460	0.2432	0.2400	0.2482	0.2383	18
C33	Smelting and pressing of non-ferrous metals	0.1376	0.1767	0.1635	0.1975	0.2478	0.2845	0.2802	0.2125	23
C34	Manufacture of metal products	0.2301	0.3091	0.2210	0.2791	0.2506	0.2524	0.2681	0.2586	16
C35	Manufacture of general purpose machinery	0.2524	0.2807	1.1100	0.4703	0.5032	0.4590	0.4599	0.5051	11
C36	Manufacture of special purpose machinery	0.2875	0.3649	0.4027	0.5061	0.6536	0.6369	1.0034	0.5507	9
C37	Manufacture of transport equipment	1.0233	1.0054	1.0810	0.6591	0.5881	0.6746	1.0055	0.8624	7
C39	Manufacture of electrical machinery and equipment	0.6520	1.0363	0.8522	1.0054	1.0014	1.0816	1.0497	0.9541	4
C40	Manufacture of communication equipment, computers and other electronic equipment	1.0173	0.7093	1.0437	1.0085	1.0681	1.0144	1.0375	0.9855	3
C41	Manufacture of measuring instruments and machinery for cultural activity and office work	0.5668	0.5675	1.0900	0.7723	1.1193	1.0699	1.0184	0.8863	6
Mean of patent-intensive manufacturing		0.4743	0.5061	0.6559	0.5572	0.6130	0.6181	0.6721	0.5852	
Mean of non-patent-intensive manufacturing		0.3819	0.3934	0.3742	0.4223	0.4399	0.4065	0.3944	0.4018	
Mean of manufacturing		0.4161	0.4351	0.4786	0.4723	0.5040	0.4849	0.4972	0.4697	

**Table 3:** Model estimation results.

Variable	Model (1)						Model (2)					
	Manufacturing		Patent-Intensive Manufacturing		Non-Patent Intensive Manufacturing		Manufacturing		Patent-Intensive Manufacturing		Non-Patent Intensive Manufacturing	
	RE	FE	RE	FE	RE	FE	RE	FE	RE	FE	RE	FE
*C*	−0.4588 (0.3771)	−1.6051 ** (0.7323)	−0.1365 (0.917)	−0.7432 (1.6995)	−0.6628 (0.4653)	−1.6903 * (0.9070)	−0.4644 (0.4031)	−1.7918 ** (0.7389)	−0.4510 (0.8861)	−2.1090 (1.8078)	−0.5656 (0.4802)	−2.0694 ** (0.8987)
*ER*	−0.1660 *** (0.0264)	−0.0866 ** (0.0360)	−0.1418 ** (0.0539)	−0.0141 (0.0716)	−0.1552 *** (0.0320)	−0.1297 *** (0.0411)	−0.1554 ** (0.0727)	−0.1289 (0.0836)	−0.3050 * (0.1617)	−0.3383 * (0.1797)	−0.0831 (0.0791)	−0.0591 (0.0939)
*IFDI*	0.1999 *** (0.0474)	0.0853 (0.0709)	0.2214 ** (0.1040)	0.0031 (0.1315)	0.1146 * (0.0611)	0.1161 (0.0844)	0.2196 *** (0.0779)	0.0663 (0.0938)	0.0964 (0.1648)	−0.3440 (0.2149)	0.2152 ** (0.0895)	0.1677 * (0.0991)
*OFDI*	0.1555 * (0.0855)	0.2386 * (0.1399)	0.0606 (0.1479)	0.0859 (0.1967)	0.1785 (0.1247)	0.3865 * (0.2057)	0.0981 (0.1185)	0.1104 (0.1587)	0.1322 (0.2119)	0.0470 (0.2652)	0.0105 (0.1560)	0.2497 (0.2156)
*CI*	−0.1920 (0.1508)	0.4341 (0.2668)	−0.1719 (0.3177)	0.2198 (0.4374)	−0.1471 (0.1939)	0.5319 (0.3624)	−0.1820 (0.1605)	0.4246 (0.2714)	−0.2807 (0.2961)	0.2465 (0.4305)	−0.1339 (0.2056)	0.6551 * (0.3679)
*SE*	0.2497 *** (0.0768)	0.0431 (0.1168)	0.2988 * (0.1615)	0.2955 (0.2065)	0.1751 * (0.1028)	−0.0479 (0.1453)	0.2498 *** (0.0793)	0.0721 (0.1177)	0.3081 ** (0.1449)	0.3292 (0.2035)	0.1906 * (0.1043)	0.0170 (0.1451)
*GS*	−0.0419 (0.0491)	−0.0431 (0.0604)	−0.0258 (0.1954)	−0.1178 (0.2360)	−0.0980 * (0.0579)	−0.0661 (0.0620)	−0.0373 (0.0497)	−0.0411 (0.0604)	−0.0658 (0.1856)	−0.2241 (0.2492)	−0.0972 * (0.0570)	−0.0676 (0.0606)
*PRS*	0.1211 * (0.0626)	−0.1282 (0.0965)	−0.0002 (0.1956)	−0.2467 (0.3664)	−0.0536 (0.0813)	−0.1356 (0.1016)	0.1072 * (0.0639)	−0.1372 (0.0969)	0.1112 (0.1702)	−0.1203 (0.3820)	0.0332 (0.0808)	−0.1298 (0.0997)
*ER × IFDI*							0.0103 (0.0237)	0.0007 (0.0253)	−0.0577 (0.0488)	−0.1074 * (0.0547)	0.0490 * (0.0266)	0.0457 (0.0288)
*ER × OFDI*							−0.0212 * (0.0259)	−0.0468 * (0.0280)	0.0324 (0.0547)	−0.0128 (0.0585)	−0.0697 ** (0.0295)	−0.0756 ** (0.0315)
ADJ-*R*^2^	0.3503	0.9082	0.3803	0.8813	0.2803	0.9182	0.3349	0.9099	0.4688	0.8899	0.3013	0.9236
*F-*statistic	13.9406	46.4906	5.4345	24.6000	6.1745	46.3844	10.0143	44.1694	5.8840	22.8920	5.2231	44.9575
Applicable model	FE		RE		RE		FE		FE		RE	
Hausman test	37.2936 ***		11.1036		\		37.9680 ***		20.3976 **		\	
Samples	189		70		119		189		70		119	

Note: The number in brackets in the table is the standard error in the coefficient estimation. ***, ** or * denotes significance at the level of 1%, 5% or 10%, respectively.

**Table 4:** Comparisons of research hypothesis and empirical results.

	Green Innovation			
	Research Hypothesis	Empirical Result		
		Manufacturing	Patent-intensive Manufacturing	Non-Patent Intensive Manufacturing
Environmental regulation (ER)	promotion (H1)	inhibition, significant	inhibition, significant	inhibition, significant
Inward foreign direct investment (IFDI)	promotion (H2)	promotion, insignificant	promotion, significant	promotion, significant
Outward foreign direct investment (OFDI)	promotion (H3)	promotion, significant	promotion, insignificant	promotion, insignificant
*ER* × *IFDI*	promotion (H4)	promotion, insignificant	inhibition, significant	promotion, significant
*ER* × *OFDI*	promotion (H5)	inhibition, significant	inhibition, insignificant	inhibition, significant
